# Perfect Zeeman Anisotropy in Rotationally Symmetric Quantum Dots with Strong Spin–Orbit Interaction

**DOI:** 10.1021/acs.nanolett.4c01247

**Published:** 2024-06-17

**Authors:** Markus Aspegren, Lila Chergui, Mikelis Marnauza, Rousan Debbarma, Jakob Bengtsson, Sebastian Lehmann, Kimberly A. Dick, Stephanie M. Reimann, Claes Thelander

**Affiliations:** † Solid State Physics and NanoLund, Lund University, SE-221 00 Lund, Sweden; ‡ Mathematical Physics and NanoLund, Lund University, SE-221 00 Lund, Sweden; § Centre for Analysis and Synthesis and NanoLund, Lund University, SE-221 00 Lund, Sweden

**Keywords:** quantum dot, quantum ring, spin−orbit interaction, symmetry, Zeeman effect

## Abstract

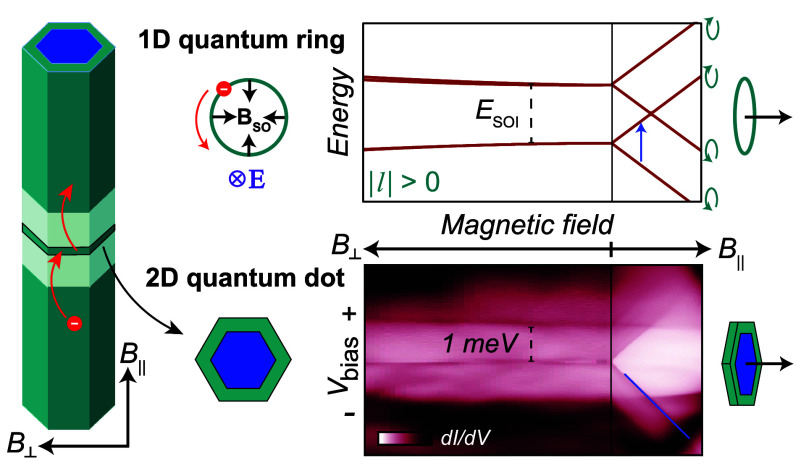

In nanoscale structures with rotational symmetry, such as quantum rings, the orbital motion of electrons combined with a spin–orbit interaction can produce a very strong and anisotropic Zeeman effect. Since symmetry is sensitive to electric fields, ring-like geometries provide an opportunity to manipulate magnetic properties over an exceptionally wide range. In this work, we show that it is possible to form rotationally symmetric confinement potentials inside a semiconductor quantum dot, resulting in electron orbitals with large orbital angular momentum and strong spin−orbit interactions. We find complete suppression of Zeeman spin splitting for magnetic fields applied in the quantum dot plane, similar to the expected behavior of an ideal quantum ring. Spin splitting reappears as orbital interactions are activated with symmetry-breaking electric fields. For two valence electrons, representing a common basis for spin-qubits, we find that modulating the rotational symmetry may offer new prospects for realizing tunable protection and interaction of spin–orbital states.

When an electron moves inside an electrostatic potential, such as under the influence of an atomic nucleus, the electron’s spin experiences an interacting magnetic field in its own rest frame. In atomic physics, this interaction between spin and orbital properties contributes to the fine structure of the spectrum. Electrons in a semiconductor crystal can be subject to spin–orbit interaction (SOI) from multiple sources, such as a noncentrosymmetric crystal host^1^ and various structural asymmetries due to confinement, interfaces, surfaces, and external electric fields.^2^ SOI is today an essential ingredient in various solid-state device concepts.^3^ In topological insulators, it is responsible for the emergence of helical edge states,^4,5^ whereas in spintronic^6^ and spin qubit applications,^7^ it provides a means to manipulate electron spins with electric fields.

In a system with rotationally symmetric confinement, such as in a quantum ring,^8−16^ the coupling of the electron’s spin to its angular momentum from SOI can give rise to a particularly strong magnetic anisotropy. If the electric field responsible for SOI is symmetric around the ring axis, then SOI will suppress Zeeman splitting of levels for any external magnetic (**B**) field oriented in the ring plane. However, a significant splitting occurs if a **B**-field component threads the orbital, which then couples to the orbital angular momentum.^17,18^ In this case, SOI opens an energy window where Lande’ *g*-factors are predicted to be an order of magnitude higher than in bulk,^19^ likely responsible for the strongly fluctuating and anisotropic *g*-factors sometimes observed in quantum dot (QD) experiments.^20−22^ By perturbing the rotational symmetry, this window closes, such that an efficient electrostatic control over the magnetic properties is possible.^14,16,19^

Although no solid-state system can attain the infinite symmetry of an ideal quantum ring, as long as the system has a periodic and rotationally symmetric potential, bands of orbital states form where orbital interactions can be completely suppressed.^10,15,23^ Carbon nanotubes are an interesting example with an inherently high radial symmetry, and where SOI emerges from the curved graphene lattice. Weak interaction of counter-propagating electron states has been observed, evidencing a low, although finite, disorder.^17,24^ However, as the electron momentum in a tubular structure is not axially restricted, electron–electron interactions and orbital separation are very different from those in a ring or a two-dimensional disk. The latter geometries were previously realized in, for example, GaAs quantum wells,^8,25^ coupled QDs of InAs,^14,15,26^ graphene,^27^ and atoms manipulated on a semiconductor surface,^13^ but limited by disorder, access to full characterization, or weak SOI.

In this work, we show that it is possible to realize an actual rotational symmetry, with no detectable disorder, inside a flat semiconductor QD. An essentially perfect Zeeman anisotropy emerges due to the combination of symmetry and strong spin–orbit interaction, where Zeeman splitting is greatly enhanced for **B**-fields threading the QD plane while fully suppressed for in-plane fields. Even when the rotational symmetry is perturbed, the presence of a strong SOI protects against orbital scattering under weak **B**-fields. With two interacting valence electrons, we find that the SOI lifts degeneracies in the ground state. Intriguingly, a comparison with a one-dimensional quantum ring model that includes Rashba SOI and Coulomb interactions allows us to identify orbital selection rules for various spin singlet–triplet interactions. The orbital degree of freedom in these QDs provides a tunable analogue to valley degrees of freedom in materials such as silicon,^28^ graphene,^29,30^ and carbon nanotubes.^17,31^ The coherence times of spin-valley qubits^32,33^ based on such materials would improve by developing a corresponding method to completely suppress the disorder parameter.

The electron orbitals in this study appear within flat semiconductor QDs, approximately 6 nm thick and 100 nm in diameter, formed along core–shell nanowires, as shown in Figure 1a–c. During growth of a zinc blende (ZB) InAs nanowire core, two segments (∼20 nm) of wurtzite (WZ) crystal structure are introduced. They provide both axial confinement and symmetry-preserving tunnel barriers to the QD for noninvasive electron transport spectroscopy.^14,34^ The core is surrounded by a ∼20 nm thick shell of InAs_1–*x*_Sb_*x*_ (*x* = 0.1–0.2),^15^ followed by a thin layer of InAs, and finally an outer, sacrificial shell of GaSb that selectively deposits on ZB and provides an indicator of the QD position.^35^ The purpose of the InAsSb shell, with a slightly reduced band gap relative to InAs, is to create a radial well where SOI is enhanced.^36^ Contacts to the nanowires are formed using electron beam lithography, followed by GaSb shell removal (5 min MF319, 5 min H_2_O), native oxide removal (15 s HCl:H_2_O 1:20), and evaporation and lift-off of 25/125 nm Ni/Au. Electron transport using DC biasing is studied at an electron temperature around 100 mK.

**Figure 1:**
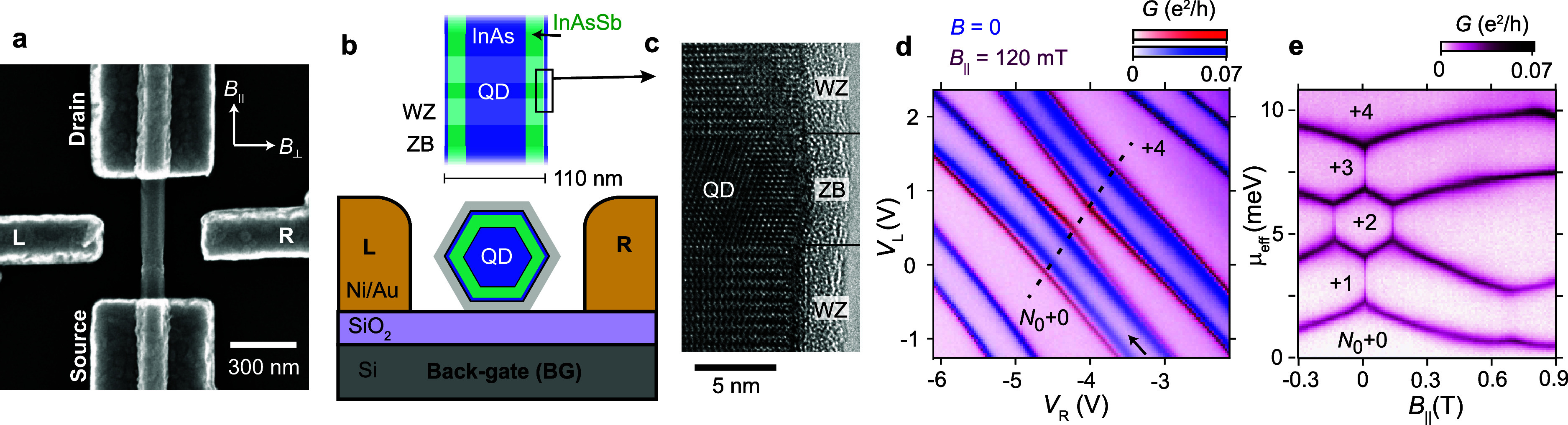
Shaping a rotationally symmetric orbital. (a) Scanning electron micrograph of a core–shell nanowire device. (b) Schematic of the axial and radial structures of the InAs–InAsSb nanowire and the device cross-section. Two tunnel barriers of wurtzite (WZ) crystal structure confine electrons to a zinc blende (ZB) QD. The outer (gray) layer indicates the sacrificial GaSb shell removed in the device processing. (c) The edge of a ZB QD and surrounding WZ barrier imaged by transmission electron microscopy (TEM) along the [101] direction. For further TEM data, see the Supporting Information. (d) Conductance measurements at *V*_BG_ = −2.4 V as a function of voltages applied to the two side gates, L and R, in panel a. The figure is an overlay of two measurements (blue, red) obtained at *B* = 0 and *B*_||_ = 0.12 T, respectively. A maximum deviation occurs at an electric field where the confinement potential is rotationally symmetric. (e) Zero-bias conductance recorded along the dashed line in panel d, showing the evolution of ground state energies with *B*_||_ as the electron population changes from *N*_0_ + 0 to *N*_0_ + 4.

We measure conductance as a function of voltages applied to the back gate (*V*_BG_) and side gates (*V*_L_ and *V*_R_) at **B** = 0, and under a **B**-field applied along the nanowire, *B*_||_ = 0.12 T. By overlaying such plots (Figure 1d), it is possible to identify electrostatic conditions where a contribution from orbital angular momentum in a rotationally symmetric potential leads to strong **B**-field induced level shifts. The pairing of lines in Figure 1d shows that the QD exhibits a clear orbital structure, where the spin degeneracy within an orbital is interrupted by the QD charging energy *E*_C_ = 1.9 meV. We note that the QD is in a many-electron regime, with an estimated electron population *N*_0_ ∼ 30 in filled orbitals. Going to such high electron numbers enhances the orbital angular momentum and thereby spin–orbit interaction.^19^ Higher orbitals are also less sensitive to small fluctuations in the underlying potential.^10^Figure 1e shows the evolution of ground state (GS) energies with *B*_||_ along the dashed line in Figure 1d, where the coupling to *B*_||_ is the strongest. In a regime of two additional electrons (*N*_0_ + 2), we find a GS transition at *B*_||_ = 0.13 T and also interactions with other orbitals near *B*_||_ ∼ 0.7 T.

We next focus on a regime where the QD hosts one unpaired spin, studied within the *N* = *N*_0_ + 1 charge configuration of Figure 1e. Inside such a regime, the total QD charge is fixed due to Coulomb blockade, but transport via excited states can still occur through inelastic cotunneling. The strongly reduced tunnel rates for such transport processes allow spectroscopy with high resolution, not limited by lifetime broadening. We find giant anisotropy in the spectroscopic data of Figure 2d, recorded as functions of *B*_||_ and a **B**-field perpendicular to the nanowire (*B*_⊥_). Even though the plotting range for *B*_⊥_ is much wider, the transition energies remain essentially constant. In order to understand the data, we compare with calculations of one-electron states in a perfect 1D quantum ring with a 60 nm diameter (see ref (16) and Supporting Information for details). In the absence of SOI, the ±*l* orbitals are degenerate at **B** = 0 as shown in Figure 2a. A *B*_⊥_ results only in an energy shift due to Zeeman spin splitting, whereas a *B*_||_ shifts the states also with a contribution from the orbital angular momentum. We can here introduce an effective Landé *g*-factor (g*) to describe the Zeeman splitting of states in a magnetic field, *E*_Z_ = g*μ_B_|**B**|, where μ_B_ is the Bohr magneton. With no SOI, g* can be expressed as the sum of spin and orbital contributions, g* = *g*_spin_^*^ + *g*_orb_^*^ cos θ, where *g*_orb_^*^ depends on orbital quantum number (±*l*) and θ is the angle of the external **B** field relative to the ring axis.

**Figure 2:**
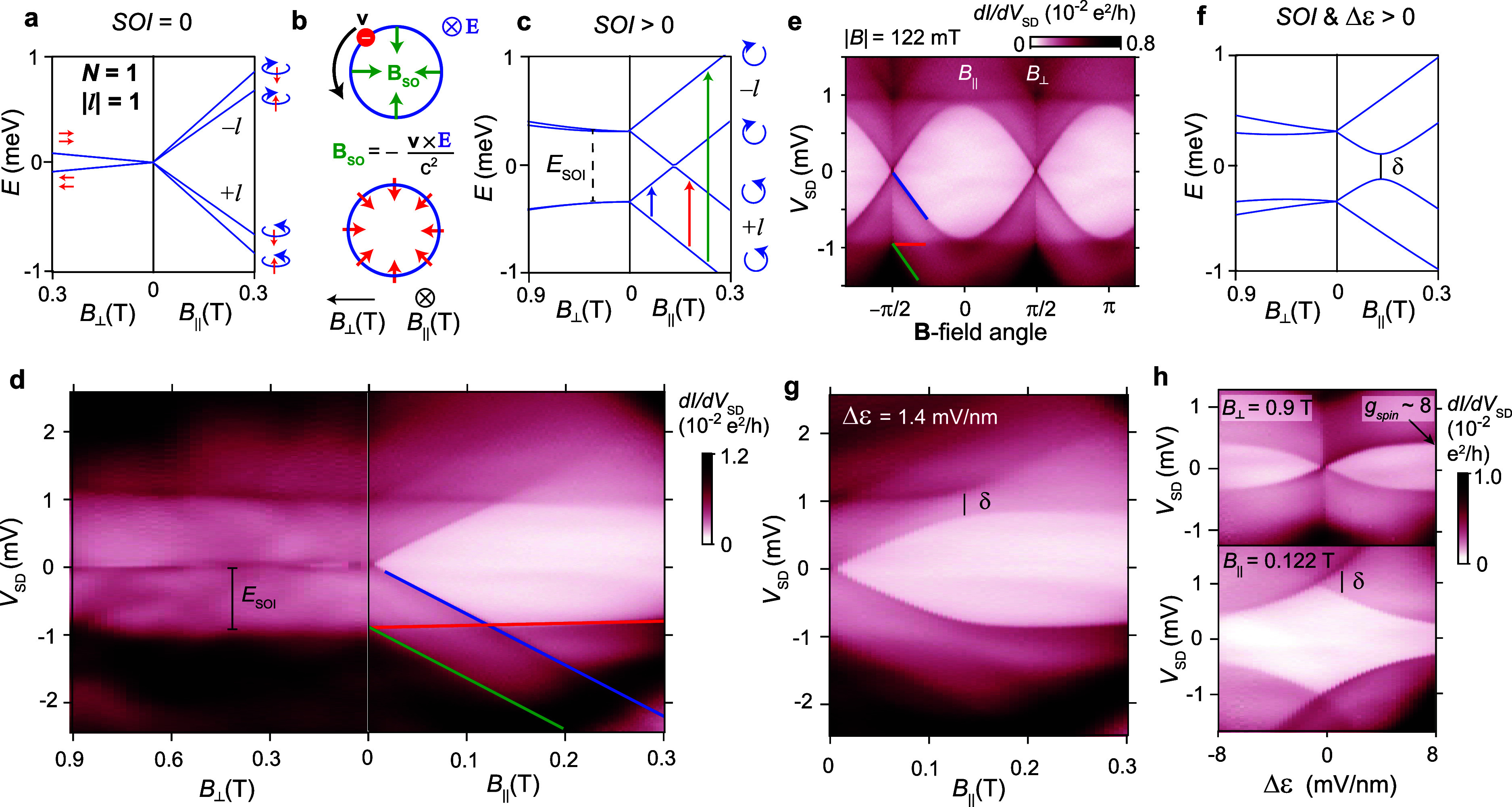
A ring-like orbital with one valence electron. (a) Calculated energies of the |*l*| = 1 orbital for a single electron (*N* = 1) in a 1D ring without spin–orbit interaction (SOI = 0). For clarity, the diamagnetic *B*^2^ term affecting all states has been omitted in all plots, and *E* (meV) is set relative to the gap center. (b) SOI from an axial **E**-field polarizes electron spins radially. (c) Corresponding calculated electron energies for SOI > 0, where a much wider plotting range is used for *B*_⊥_. (d) Cotunneling transport spectroscopy in the *N* = *N*_0_ + 1 configuration of Figure 1d. A darker contrast indicates that a transition involving an excited state is possible. The colored lines correspond to the different transitions indicated in panel c. (e) Effect of rotating a **B**-field, |**B***|* = 122 mT, in the sample plane. A maximum splitting of states occurs when **B** is aligned with the nanowire (*B*_||_), and a total suppression occurs when the relative angle is π/2 (*B*_⊥_). (f, g) A change in the **E**-field in the orbital plane breaks the symmetry and introduces orbital interaction as shown by calculations in panel f and the experiment in panel g. (h) Data obtained along the *N*_0_ + 1 electron regime indicated by the arrow in Figure 1d, going from [*V*_L_; *V*_R_] = [−1.48; −2.94] to [2.07; −6.10] V, where a linear **E**-field is assumed. At *B*_⊥_= 0.9 T in the upper panel of panel h, Zeeman splitting is possible for any Δε ≠ 0. The lower panel shows the corresponding measurement at *B*_||_ = 0.122 T where orbitals with different signs of *l* come close in energy and where scattering is absent for Δε = 0.

When introducing a Rashba SOI (Figure 2b), a splitting of the Kramers pairs occurs as shown in Figure 2c. Assuming an axial electric field, such as from spontaneous polarization charge in the wurtzite tunnel barriers,^37^ the spin–orbit field (**B**_SO_) induced by SOI is radial, leading to a radial spin-polarization.^11,38,39^ All level splitting in Figure 2d is therefore suppressed along *B*_⊥_ due to the large |**B**_SO_| ∼ 1.7 T, assuming *E*_SOI_ = 1 meV and *g*_spin_^*^ = 10. We note that a similar suppression along *B*_⊥_ is expected also in the case of a radial **E**-field, which instead provides an axial spin polarization.^17^ Along *B*_||_, the two cases differ though, where the coupling to the spin magnetic moment is initially suppressed if **B**_SO_ is radial. Experimentally, it is difficult to distinguish between the two cases since the contribution from *g*_spin_^*^ is very small compared to that from *g*_orb_^*^.

Small level variations near zero bias are visible along *B*_⊥_ in Figure 2d, which we partly attribute to limitations in controlling the angle of the external magnetic field. This angular sensitivity is obvious in Figure 2e, where an external **B**-field, |*B*| = 122 mT, is rotated in the sample plane and where splitting is suppressed only for an exact π-periodic angle, where no magnetic flux threads the orbital. The horizontal features (red line) here give an indication of the SOI-induced gap. After compensating for series resistances in the setup during cotunneling measurements (1 MΩ), we extract *E*_SOI_ = 0.9 meV and *g*_orb_^*^ = 115, corresponding to an orbital magnetic moment, |**μ**| = 58 μ_B_. The Supporting Information provides an example of another orbital in the same QD with *E*_SOI_ = 1 meV and *g*_orb_^*^ = 160 (|**μ**| = 80 μ_B_).

Any change in the electric (**E**) field in the ring plane perturbs the symmetries in the confinement potential, which results in orbital interactions and lower orbital angular momentum. However, we note from Figure 2f that SOI prevents orbital mixing for weak *B*_||_ assuming that the disorder (δ) is moderate (quantified by the avoided orbital crossing). Figure 2g shows an experimental example of this situation, where the system is detuned from the optimum symmetry point, corresponding to a change in the **E**-field of Δε = 1.4 mV/nm assuming a linear field. Since *E*_SOI_ > δ, the effect of disorder remains small for sufficiently weak *B*_||_.

Figure 2h demonstrates the corresponding effect of detuning when instead fixing the **B**-field and varying the **E**-field. The data were similarly recorded in the *N*_0_ + 1 regime but along the diagonal stripe of constant charge indicated by the arrow in Figure 1d. In the lower panel, an applied *B*_||_ = 0.122 T, has brought states with opposite signs of *l* close in energy, where an exact crossing appears only for a specific electrostatic condition where interaction is fully suppressed. Away from this point, the symmetry is broken, and orbital mixing is possible. The upper panel of Figure 2h, recorded at *B*_⊥_= 0.9 T, shows the opposite effect of detuning on the Zeeman energy. Zeeman splitting is here completely suppressed when the system has rotational symmetry, whereas |g*| under detuning eventually recovers to a value close to the typical one for InAs QDs, |*g*_spin_^*^| ∼ 8. We note that the effects of a change in **E**-field on Zeeman splitting show a good agreement with predictions in ref (19) for orbitals in InAs nanowires. The data also support the findings in ref (14) that an orbital in a single QD is relatively robust against detuning, in contrast to a ring composed of two coupled QDs, where the electron becomes strongly localized.

Adding a second valence electron to the |*l*| > 0 orbital pair provides us with an opportunity to study electron–electron interactions in a system with rotational symmetry.^9,40^ In a typical QD with no particular symmetries and a weak SOI, the ground state is generally a spin-singlet. However, if the valence orbitals are energetically close, the spin-triplet can become favored (Hund’s rule) as it minimizes the Coulomb interaction.^16,29,41,42^

Figure 3a–c show results from calculations for an ideal 1D quantum ring with *N* = 4 electrons, where two electrons reside in the *l* = 0 orbital (filled) and two in |*l*| = 1 (half-filled). Assuming a sufficiently large orbital separation, similar behavior should be repeated for any *N* being a multiple of 4, where the valence orbital pair (±*l*) is half-filled. In the following, we disregard electrons in filled orbitals and use the term “two-electron states” to describe the system. Due to the rotational symmetry, six different two-electron states can occupy an |*l*| > 0 orbital: three singlets and three triplets. Two of the singlets stand out as they have a nonzero orbital angular momentum, with both spins in the same orbital. The six states are all degenerate in the case of no Coulomb interaction and SOI as shown in Figure 3a. When Coulomb interactions are included in an exact diagonalization approach (Figure 3b), a singlet–triplet splitting occurs, where the triplets become ground state in agreement with Hund’s rule, and we note also a splitting among the singlets. This particular ground state was recently found in a similar system with a ring composed of two coupled QDs, but where SOI was much weaker than the exchange energy.^16^

**Figure 3:**
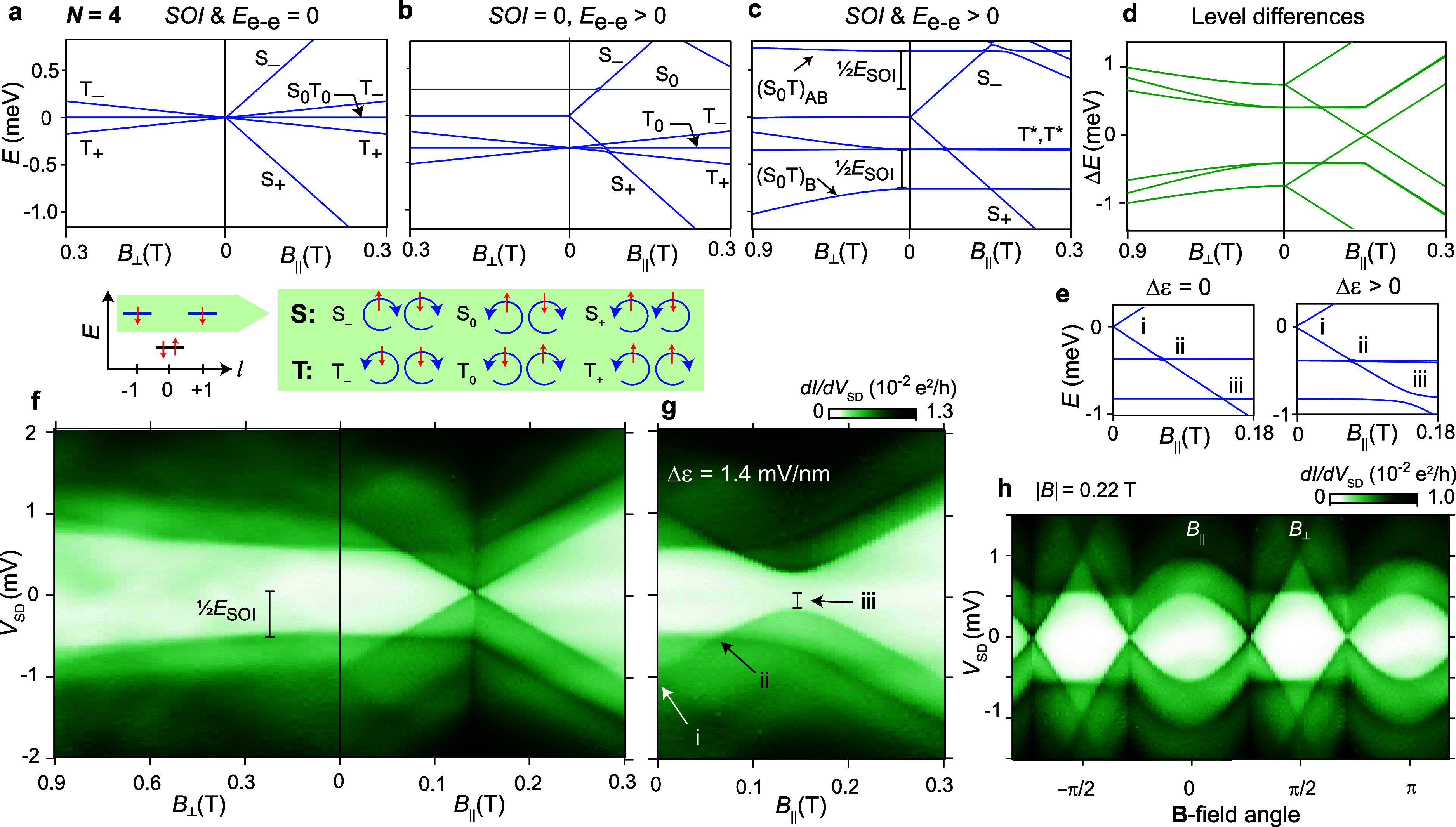
Two valence electrons. (a–c) Calculated energy levels of a quantum ring with *N* = 4 electrons. For clarity, the diamagnetic *B*^2^ term affecting all states has been omitted (see Supporting Information), and *E* (meV) is set relative to *E* of S_±_ at *B* = 0. The two valence electrons in the |*l*| = 1 orbitals form six two-electron states. Panel a is without interactions, panel b with Coulomb (*E*_e–e_) interactions, and panel c also includes SOI. The lines at higher energy, coming down with *B*_||_, correspond to excited states with only one electron in |*l*| = 0 and three electrons in |*l*| = 1. (d) Calculated level differences showing low-energy transitions involving the ground state based on panel c. (e) Effect of orbital detuning in the model. (f) Cotunneling transport spectroscopy of the *N* = *N*_0_ + 2 regime, which basically replicates the calculations in panel d. (g) Effect of orbital detuning. An avoided crossing is observed only in point iii, where S_+_ can interact with the S_0_ component of the SOI-hybridized S_0_T bonding state. (h) Rotation of a **B**-field, |**B***|* = 220 mT, in the sample plane for Δε = 0.

When SOI is introduced (Figure 3c,d), we cannot apply Hund’s rule as the two-electron spin states are no longer pure.^18,24,43^ However, as we will show, some states remain relatively pure due to their unique orbital character. At **B** = 0, Figure 3c indicates that one state splits off from the triplets relative to Figure 3b due to SOI, such that the ground state becomes nondegenerate. The primary cause for this interaction is the S_0_ state, which becomes perturbed correspondingly and is pushed to a higher energy. We also note that spin splitting of states is suppressed due to the radial **B**_SO_.

A spectroscopic measurement of the regime with two valence electrons (*N*_0_ + 2) is shown in Figure 3f, plotted for *B*_⊥_ and *B*_||_. Here, we find the predicted absence of a degenerate ground state at *B* = 0 and the presence of a ground state change at *B*_||_ = 0.14 T. If |**B***|* > 0.14 T is rotated in the sample plane (Figure 3h), the amount of magnetic flux picked up by the S_+_ orbital determines whether the ground state changes. By comparing Figure 3f with the calculated level differences plot in Figure 3d, we can identify all predicted transitions involving the ground state, except with the S_0_–T antibonding state pushed to high energy. As predicted by the model, the *E*_SOI_ extracted from Figure 3f correlates with the corresponding *E*_SOI_ in the one-electron regime in Figure 2d.

We find it remarkable that a few-electron and few-parameter 1D model can so accurately describe the physics of such a complex experimental many-electron system. The dielectric constant is a particularly important fitting parameter, here increased by a factor of ∼10 compared to the InAs bulk value, motivated by the approximately 10 times higher *N* in the experiment, as well as screening from electrons in the source and drain. We also note that the orbital angular momentum of the valence electrons in the model and experiment are rather similar, despite the very different number of electrons, *N*, in the sample. We believe this is partially a consequence of not having infinite rotational symmetry as in the model. Even though orbital interaction is fully suppressed due to symmetry, the orbital angular momentum will still be reduced by the presence of a periodic potential.^10^ Moreover, we cannot rule out the possibility of radial nodes in the wave function, which would allow for states with lower orbital angular momentum at correspondingly higher *N*.

By activating orbital mixing through detuning, as shown in Figure 3e,g, we can confirm the nature of the spin–orbital states and how they interact. In point i, we find only a small interaction between S_+_ and S_–_, which couple by a second order process where both electrons change orbit, thereby suppressing hybridization. In point ii of Figure 3g, there is no visible interaction of S_+_ with either of the two T* states. This indicates that these latter states do not have significant S_0_ intermixing. Finally, in point iii, we find a strong interaction where the ground state changes. Here, S_+_ can hybridize through orbital interaction with the S_0_ component of the bonding level of the S_0_T hybrid.

We note that even though **B**_SO_ is radial and therefore perpendicular to an axial magnetic field (*B*_||_), spin–orbit mixing between states with different orbital angular momenta is suppressed due to symmetry. As shown in the Supporting Information, even when changing the external **B**-field direction away from the ring axis, such that interaction via SOI should be enhanced in points ii and iii, almost no splitting is found due to the different orbital character of the states. This indicates a tunable orbital protection from spin-flips and also highlights a possible spin–orbital analogy to spin-valley physics studied in materials with valley degrees of freedom, such as silicon,^28^ graphene,^29,30^ carbon nanotubes,^17,31^ and transition metal dichalcogenides.^44,45^

In conclusion, we have shown that a quantum dot with a rotationally symmetric potential and SOI has tunable and strongly direction-dependent magnetic properties. By creating and breaking the symmetry, the effects of SOI and its interplay with external **B**-fields can be controlled. For one valence electron, we demonstrated perfect suppression of the Zeeman effect for in-plane **B**-fields, which was gradually lifted by the introduction of orbital scattering. For two valence electrons, we found that interactions requiring a change in both spin- and orbital-angular momentum are strongly suppressed, even under the presence of weak symmetry breaking. Similar physics as observed here should appear also in other two-dimensional QDs with rotational symmetry. Recent measurements on graphene QDs have shown exceptionally large orbital magnetic moments.^27^ Even though SOI is intrinsically very weak in graphene, it can be proximity-induced from interfacing materials^46^ and thereby provide a new regime for studies and exploitation of spin–orbit interaction.

## Supporting Information Available

Nanowire epitaxy conditions and structural analysis, description of the theoretical model and additional electrical measurements (PDF)
