# Supplementary material for: Perfect Zeeman Anisotropy in Rotationally Symmetric Quantum Dots with Strong Spin–Orbit Interaction
Source: Nano Lett. 2024 Jun 17;24(26):7927–33. doi: 10.1021/acs.nanolett.4c01247 (PMC11229058; doi:10.1021/acs.nanolett.4c01247)
Supplement: Supplementary file 1 — nl4c01247_si_001.pdf [file nl4c01247_si_001.pdf]

Supplementary Information
Perfect Zeeman anisotropy in rotationally symmetric quantum dots with strong spin-orbit interaction

Markus Aspegren[1], Lila Chergui[2], Mikelis Marnauza[3], Rousan Debbarma[1], Jakob Bengtsson[2], Sebastian Lehmann[1], Kimberly Dick[3], Stephanie Reimann[2], and Claes Thelander[1]
[1]*Solid State Physics and NanoLund, Lund University, SE-221 00 Lund, Sweden*
[2]*Mathematical Physics and NanoLund, Lund University, SE-221 00 Lund, Sweden and*
[3]*Centre for Analysis and Synthesis and NanoLund, Lund University, SE-221 00 Lund, Sweden*

NANOWIRE EPITAXY AND TRANSMISSION ELECTRON MICROSCOPY

InAs nanowires with controlled crystal structure were grown by metal-organic vapor phase epitaxy (MOVPE) in an AIXTRON 3 × 2" close coupled showerhead reactor. Before growth, arrays of Au pads were defined on InAs 111B substrates by electron beam lithography. Annealing was carried out at a set temperature of 550 °C under AsH_3/H_2. Nanowire growth was initiated at 460 °C, by introducing trimethylindium (TMIn) at a molar fraction of χ_{TMIn} = 2.7E-6 and lowering the AsH_3 molar fraction. The crystal phase switching was realized by changing the AsH_3 molar fractions from $\chi_{\mathrm{AsH_3}}$ = 2.5E-2 for ZB growth to $\chi_{\mathrm{AsH_3}}$ = 2.2E-5 for WZ growth. The growth times were 360 and 240 s for the bottom and top ZB segments, respectively, and 26/10/20 s for the WZ/ZB/WZ quantum well structure. After the axial growth, an $InAs_{1\text{-}x}Sb_x$ - InAs - GaSb sequence of radial shells was grown. $InAs_{1\text{-}x}Sb_x$ was grown for 8 minutes at 440 °C with corresponding molar fractions of χ_{TMIn} = 2.6E-6, χ_{TMSb} = 3.1E-4, $\chi_{\mathrm{AsH_3}}$ = 1.3E-4 while InAs and GaSb were deposited at 460 °C for 1 and 40 minutes, respectively, and with molar fractions of χ_{TMIn} = 1.9E-6, $\chi_{\mathrm{AsH_3}}$ = 2.5E-4 and χ_{TMGa} = 2.7E-5, χ_{TMSb} = 3.1E-5. Growth was terminated by cooling down in H_2.

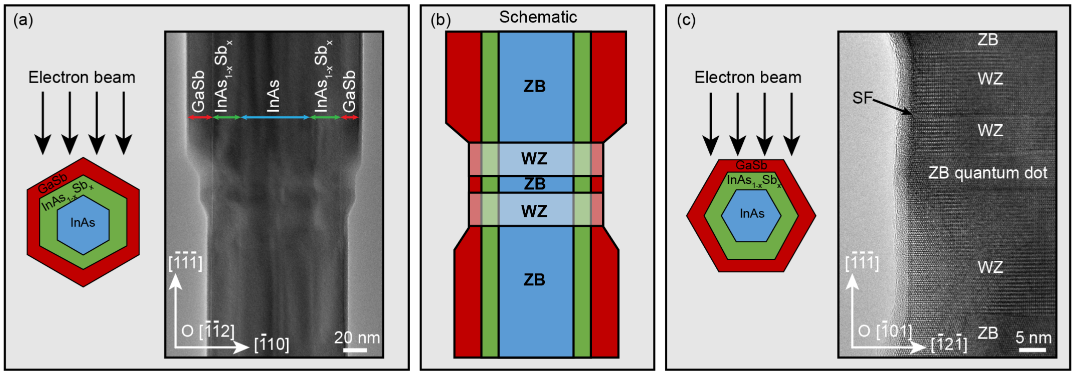

Supplementary FIG 1. Nanowire heterostructure viewed at different zone axes. (a) High-resolution transmission electron microscopy (HRTEM) image of a nanowire tilted to the zone axis where the core-shell heterostructure is visible. The schematic depicts the nanowire morphology relative to the electron beam with arrows in the image serving as a guide to the eye depicting each layer. **(b)** Schematic of the nanowire core-shell heterostructure at the quantum dot. **(c)** HRTEM micrograph of the same nanowire as in (a) tilted 30 degrees in the direction to the zone axis showing the quantum dot region of the nanowire. We note that a single stacking fault (SF) is present in the upper WZ segment of the nanowire. The schematic depicts the nanowire morphology relative to the electron beam.

1D RING MODEL

We follow to a large extent the model outlined in the supplemental material of Ref. [1] with the addition of a Rashba spin-orbit interaction term not included in that work. We model N electrons confined to a one-dimensional ring of radius R in the xy-plane. The N electrons are subject to a constant and uniform applied magnetic field $\mathbf{B} = (0, B\sin\theta, B\cos\theta)$ in the yz-plane with an angle θ from the z-axis. We take particular interest in the cases of magnetic fields applied parallel ($\mathbf{B}_{||} = B\hat{z}$) and perpendicular ($\mathbf{B}_{\perp} = B\hat{y}$) to the nanowire (the axis that threads the

ring). The system Hamiltonian for N electrons is

$$\hat{H} = \sum_{n=1}^{N} \left[\hat{h}(\varphi_n) + \hat{h}_{\mathrm{SOI}}(\varphi_n)\right] + \sum_{n>m} V_{e-e}(\varphi_n, \varphi_m), \tag{1}$$

where $\hat{h}(\varphi_n)$ is the one-body Hamiltonian without spin-orbit interaction, $\hat{h}_{\mathrm{SOI}}(\varphi_n)$ is the one-body Rashba spin-orbit interaction and $V_{e-e}(\varphi_n, \varphi_m)$ is the Coulomb interaction potential between two electrons at azimuthal angles φ_n and φ_m as given in the supplemental material of Ref. [1]. The one-body Hamiltonian is

$$\hat{h}(\varphi_n) = -\frac{\hbar^2}{2m^* R^2}\frac{\partial^2}{\partial \varphi_n^2} - i\hbar\frac{eB\cos\theta}{2m^*}\frac{\partial}{\partial\varphi_n} + \frac{e^2B^2R^2}{8m^*}(\sin^2\varphi_n\cos^2\theta + \cos^2\varphi_n) + \frac{g^*_{\mathrm{spin}}\mu_B}{2}\boldsymbol{\sigma}_n \cdot \mathbf{B} + V(\varphi_n). \tag{2}$$

Here $\hbar$ is the reduced Planck constant, m^* is the effective electron mass, $\boldsymbol{\sigma} = (\sigma_z, \sigma_y, \sigma_z)$ is the Pauli vector, g^*_{spin} is the effective electron spin g-factor, $\mu_B = e\hbar/(2m_e)$ is the Bohr magneton with bare electron mass m_e, and

$$V(\varphi) = V_g \cos(\varphi) \tag{3}$$

is a linear tilting potential, such as from an applied side-gate voltage, of strength V_g. For parallel applied magnetic fields ($\theta = 0$) the B^2 term of Eq. (2) is a uniform parabolic shift to all energy levels. For a perpendicular magnetic field the B^2 term provides both a parabolic shift in energy levels, and an effective squeezing of the ring [2]. In Figures 2 and 3 of the main article we neglect the B^2 term in the theoretical subfigures. For completion we include the effects of this term in Supplementary Figs. 2-4.

The Rashba SOI for electrons confined to a one-dimensional ring takes the form

$$\hat{h}_{\mathrm{SOI}}(\varphi_n) = \alpha\frac{eBR\cos\theta}{2}\Big[\cos\varphi_n\sigma_x + \sin\varphi_n\sigma_y\Big] - \alpha i\frac{\hbar}{R}\Big[\sigma_x\Big(\cos\varphi_n\frac{\partial}{\partial\varphi_n} - \frac{1}{2}\sin\varphi_n\Big) + \sigma_y\Big(\sin\varphi_n\frac{\partial}{\partial\varphi_n} + \frac{1}{2}\cos\varphi_n\Big)\Big] \tag{4}$$

where α is the SOI coupling strength [3]. ($\alpha \to \alpha \cdot 10^{-9} \cdot e/\hbar$ to obtain the SOI coupling strength in units of eV·nm.) The first term of Eq. (4) is dependent on the applied magnetic field and the second term is independent of the applied magnetic field.

As in Ref. [1] we use $m^* = 0.023m_e$ and $g^*_{\mathrm{spin}} = 10$. We take R, ϵ_r, and α to be fitting parameters of the model and find that $R = 30$ nm, $\epsilon_r = 150$, and $\alpha = 0.035$ eV·nm provide the best fit with the experimental data presented in Figures 2 and 3 of the main article. For the relative permittivity of the nanowire $\epsilon_r = 150$ is approximately a factor of 10 larger than the bulk value of InAs. We attribute this factor to the screening effects of closed shell electrons, as well as of nearby electrons outside of the system. We compute the spectra for $N = 1$, and $N = 4$ corresponding to a single valence electron and two valence electrons respectively. For calculations that include detuning (denoted $\Delta\varepsilon > 0$ in the main text) $V_g = 1.0$ meV was used, otherwise $V_g = 0$.

Following the procedure of the supplementary material of Ref. [1] we diagonalize the Hamiltonian Eq. (1) using the configuration interaction (CI) method in a B-spline setup. We first find the spatial eigenstates and corresponding eigenenergies of Eq. (2), which form the spatial one-body basis. These eigenstates are constructed from 500 B-splines of fifth-order, with knot points distributed linearly around the one-dimensional ring. The one-body basis functions are constructed for zero applied magnetic field. We truncate the resulting one-body basis, keeping only the 30 lowest energy spatial one-body states. The many-body basis is constructed from properly symmetrized products of the one-body spatial- and spin-states. The resulting Hilbert space is truncated by excluding all many-body states with energy greater than the energy of the state corresponding to the non-interacting ground state with a single electron excited to the first excluded one-body orbital. Finally, we diagonalize Eq. (1) in the resulting Hilbert space. For further details on the computational procedure see the supplementary material of Ref. [1].

EXTENDED PLOTTING RANGES FROM CALCULATIONS OF 1D QUANTUM RING STATES

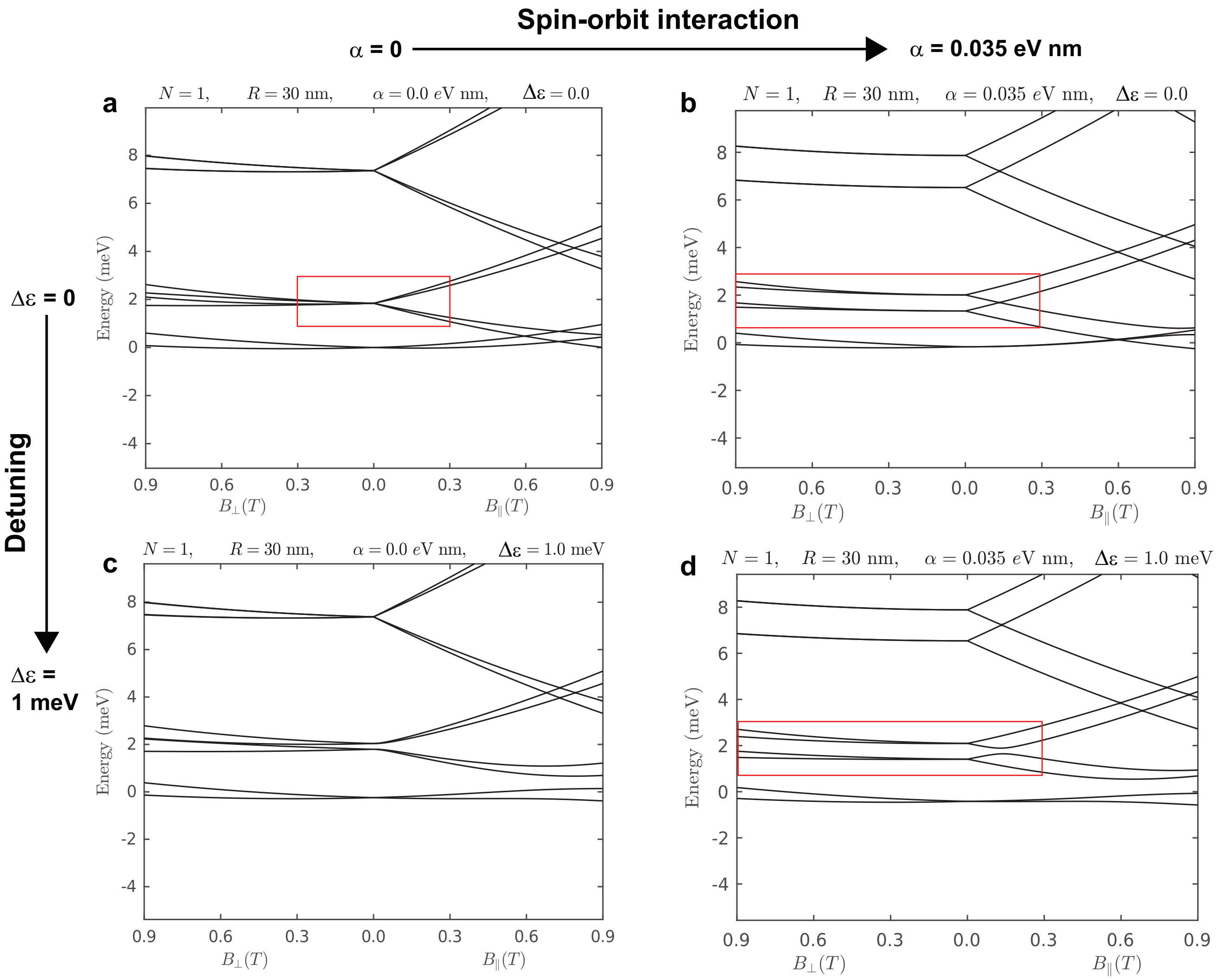

Supplementary FIG 2. Energies for $N = 1$ states involving the $l = 0, \pm1, \pm2$ orbitals (at $B = 0$) with increasing $B_\perp$ and $B_{||}$ for a 1D quantum ring with 60 nm diameter. The calculation includes the diamagnetic B^2 term excluded in the main article, which causes a parabolic shift of all levels and a symmetry breaking effect for large $B_\perp$. The red rectangles indicate the approximate plotting ranges found in the main article. **(a)** No SOI or detuning. **(b)** SOI ($\alpha = 0.035$ eV·nm) included. **(c)** A linear detuning of 1 meV included, which causes orbital interactions that primarily affect the $|l| = 1$ states (the $|l| \geq 2$ states have a much higher kinetic energy). **(d)** Both SOI and detuning.

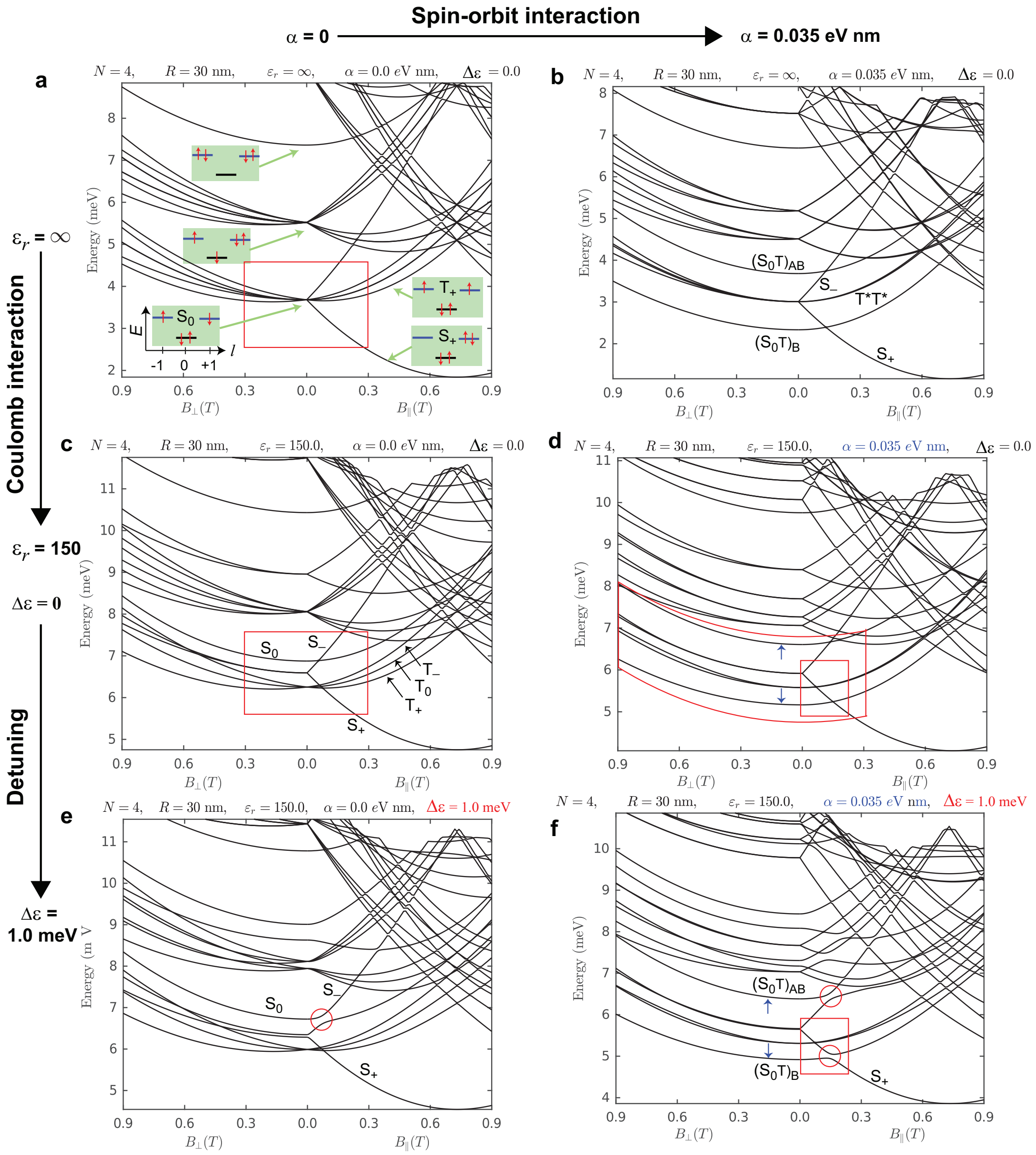

Supplementary FIG 3. Energies for $N = 4$ states involving primarily (for weak B-fields) the $l = 0, \pm 1$ orbitals, plotted against $B_\perp$ and $B_{||}$. The calculation includes the diamagnetic B^2 term excluded in the main article, which causes a parabolic shift of all levels and a symmetry breaking effect for large $B_\perp$. The red rectangles indicate the approximate plotting ranges found in the main article. **(a)** No interactions. **(b)** SOI ($\alpha = 0.035$ eV·nm) included. **(c)** Coulomb interactions included, where the large dielectric constant ($10 \times \epsilon_r$ InAs) is a fitting parameter to account for the strong screening in the experiment. The triplets become ground state at $B = 0$ (Hund's rule), and there is also a split among the three singlets. **(d)** Both SOI and Coulomb interactions. The primary interaction is between S_0 and the triplets (same orbital configuration), where two states are repelled; the bonding (B) and antibonding (AB) levels of S_0T. The interaction thus seem to be collected into one triplet, where the other two triplets are not shifted relative to the S+ and S-. **(e)** Detuning and Coulomb interaction. S- can now interact with S_0 through orbital interaction (red circle). However, S+ cannot interact with the triplets without SOI. **(f)** SOI, detuning and Coulomb interaction. Now S+ can interact with $(S_0T)_B$ via the singlet component (red circle), but there is no visible interaction with T* and T* where both spin-flip and orbital-flip is required. Even though $B_{||}$ is perpendicular to the radial B_{SO}, spin-flipping via SOI is suppressed due to the near-symmetry conditions.

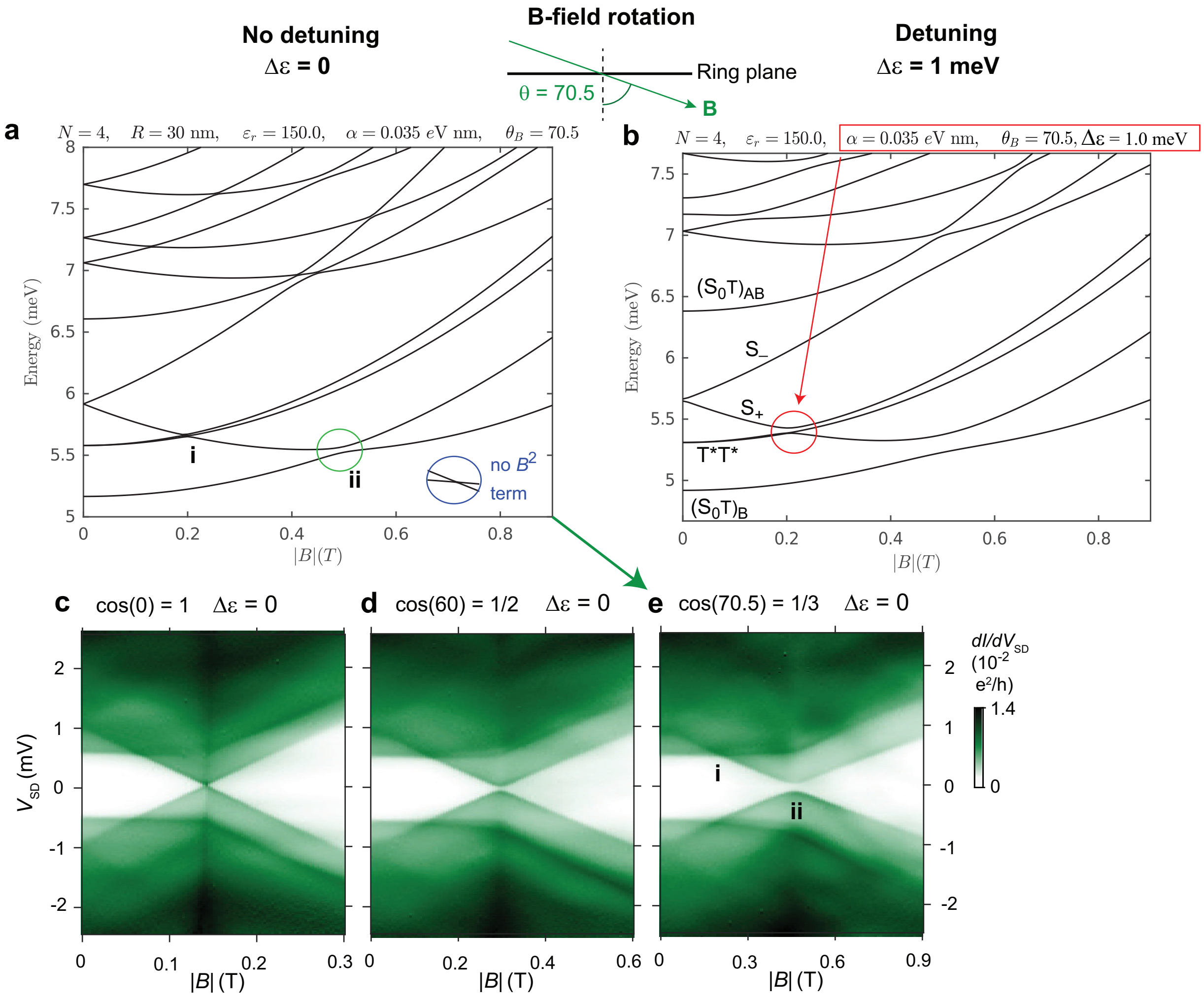

Supplementary FIG 4. **(a)**,**(b)** Calculated electron energies for a ring with $N = 4$ for B-fields applied at an angle 70.5° relative to the ring axis, such that the flux that threads the ring is $\cos(70.5) = 1/3$ relative to $B_{||}$ fields. (a) includes SOI and Coulomb interactions, and (b) also includes detuning. In (a), we note that there is no visible interaction in point i, whereas a small interaction occurs in point ii (green circle). This latter interaction is not an effect of SOI, but a consequence of symmetry breaking from a significant $B_\perp$ component which squeezes the wave function and effectively creates two QDs coupled into a ring, thus giving rise to orbital mixing. If the same calculation is done without the B^2 term, the corresponding crossing is exact (blue circle/inset). In (b), when all ingredients are present (SOI, B-field rotation, detuning), it becomes possible to resolve interactions (red circle) between S+ and the triplets. **(c)**, **(d)**, **(e)** Cotunneling spectroscopy in the $N = N_0 + 2$ regime for $\Delta\varepsilon = 0$ (zero detuning), showing the effect of external magnetic field direction for angles of 0° ($B_{||}$), 60° and 70.5° relative to the nanowire axis. We note a good agreement between panels (a) and (e). Unfortunately, the situation in panel (b) that also includes detuning was not studied experimentally.

ADDITIONAL DATA FOR THE RING-LIKE ORBITAL AT $V_{BG} = -2.4$ V

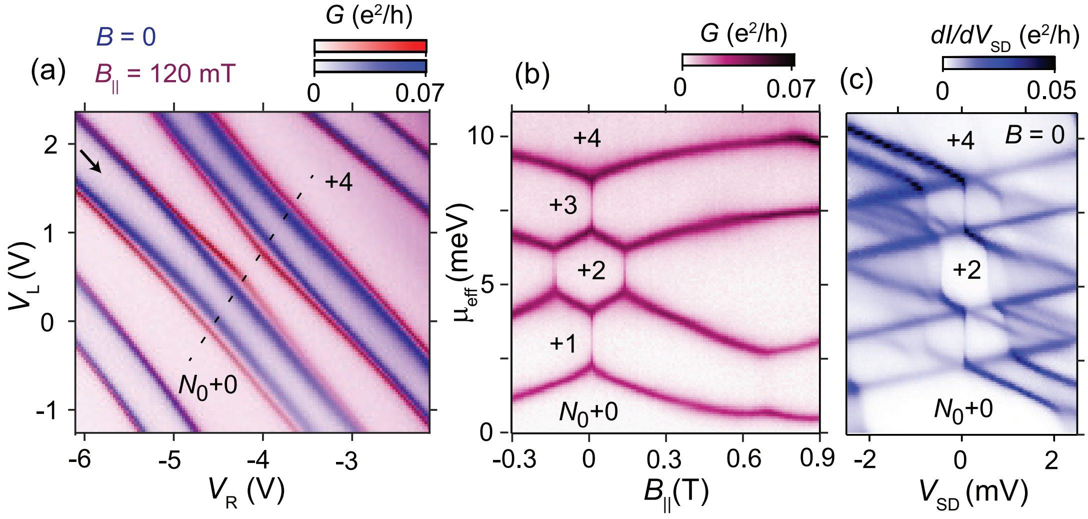

Supplementary FIG 5. Shaping a rotationally symmetric orbital. The first two figures are repeated from Fig. 1(d),(e). **(a)** Conductance measurements at $V_{\mathrm{BG}} = -2.4$ V as function of voltages applied to the two side-gates. The figure is an overlay of two measurements (blue, red) obtained at $B = 0$ and $B_{||} = 0.12$ T respectively. A maximum deviation occurs at an electric field where the QD potential has rotational symmetry. **(b)** Zero-bias conductance recorded along the dashed line in panel (a), showing the evolution of ground state energies with $B_{||}$ as the electron population changes from $N_0 + 0$ to $N_0 + 4$. **(c)** Differential conductance (dI/dV_{SD}) recorded as a function of source-drain voltage, V_{SD}, along the dashed line in panel (a). The measurement reveals a series of Coulomb blockade diamonds, which correspond to successive filling of the QD with additional electrons. Outside Coulomb blockade, transport takes place through sequential electron tunneling, where a line indicates an excited state falling inside the V_{SD} bias window. From the height of the odd occupation diamonds we extract a QD charging energy, $E_C \approx 1.9$ meV.

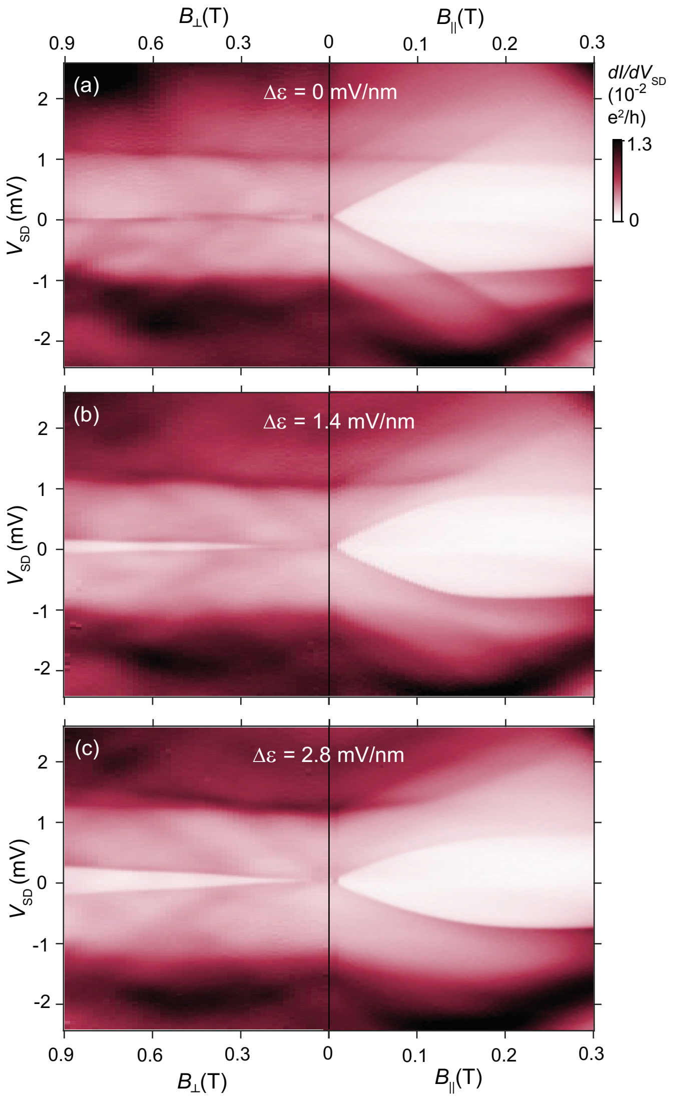

Supplementary FIG 6. Effect of orbital detuning and mixing on Zeeman splitting in the $N = N_0 + 1$ electron regime. **(a)** Cotunneling transport spectroscopy in the N_0+1 configuration in Fig. 1(c) at zero detuning. A darker contrast indicates that a transition involving an excited state is possible. **(b,c)** A detuning electric field breaks the rotational symmetry and introduces orbital scattering. The disorder provides a window for Zeeman spin splitting along $B_\perp$.

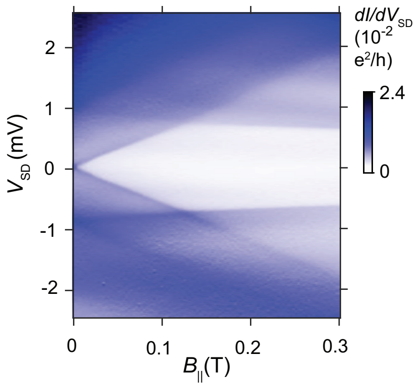

Supplementary FIG 7. Cotunneling spectroscopy of the ring-like orbital in the $N = N_0 + 3$ regime.

DATA FOR A SECOND RING-LIKE ORBITAL FORMING AT $V_{\mathrm{BG}} = -0.9$ V

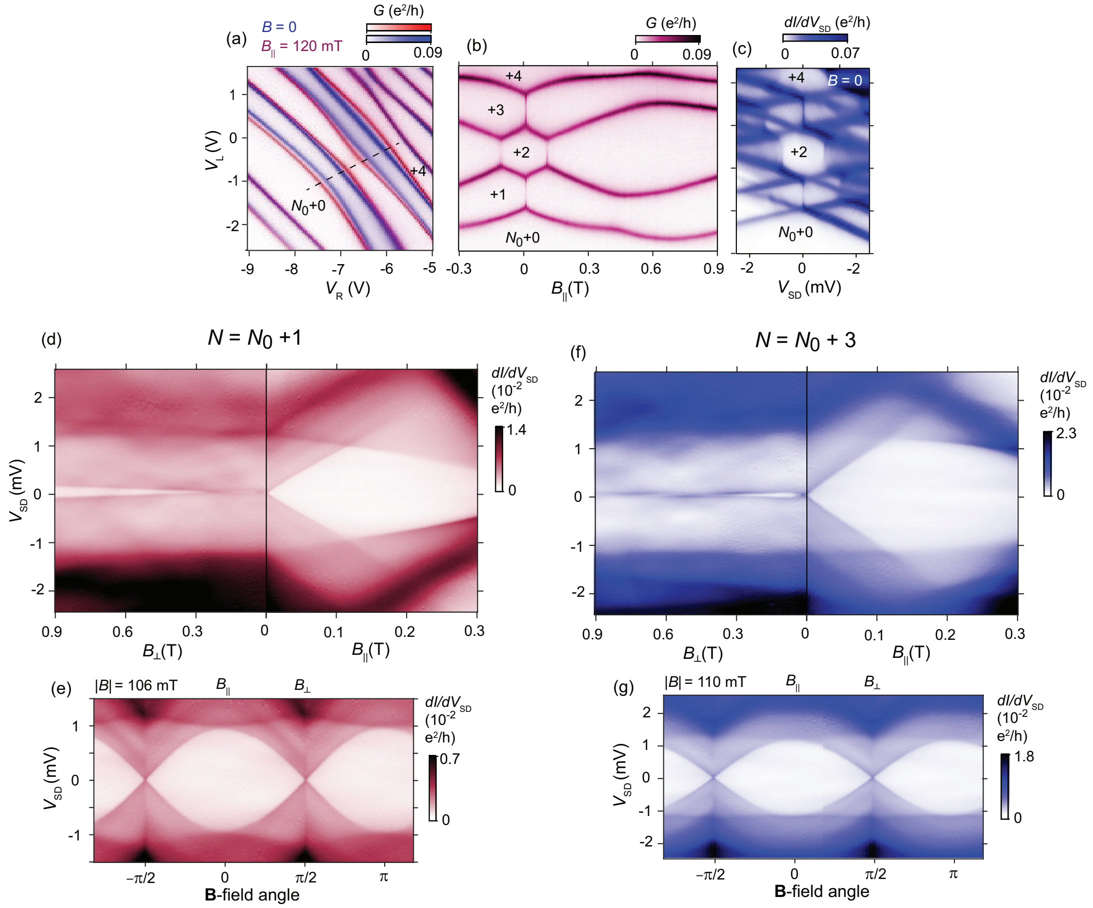

Supplementary FIG 8. Formation and characterization of a second ring-like orbital. (a-c) Similar characterization as in S1. However, due to a large series resistance in these measurements, the conductance (G) provided in the legends are here estimates. **(d,e)** Cotunneling spectroscopy in the $N = N_0 + 1$ regime for different external B-field directions. In panel (d) we note a strong decrease in the SOI-related gap along $B_{||}$. This either points to a very large $|g^*_{\mathrm{spin}}| \approx 35$, but is more likely an indication that one of the states involved in the transition mixes with another state, thereby modifying the orbital angular momentum. **(f,g)** Corresponding data in the $N = N_0 + 3$ regime.

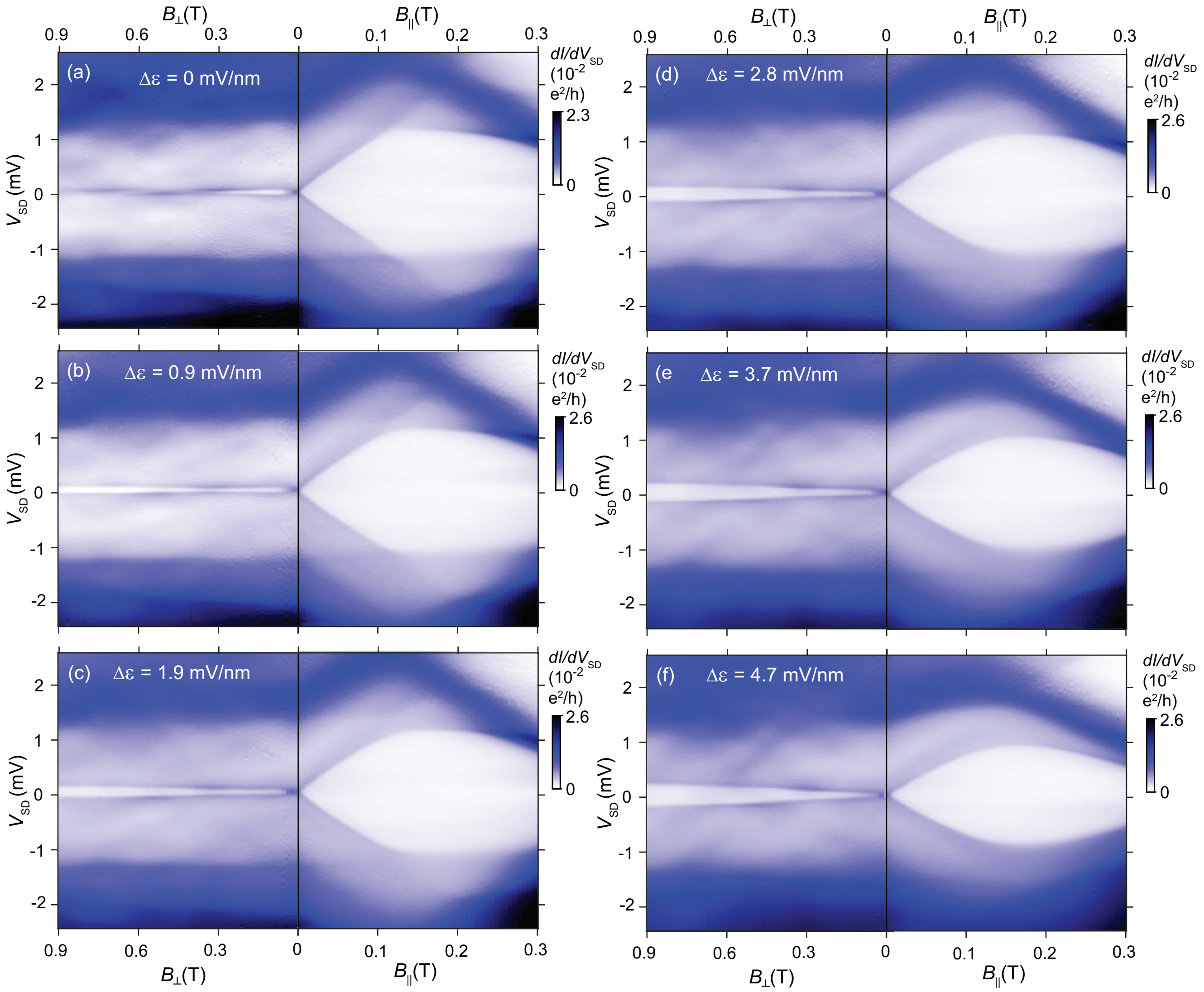

Supplementary FIG 9. Effect of orbital detuning on the Zeeman splitting in the $N = N_0 + 3$ electron regime. **(a-f)** Cotunneling transport spectroscopy in the $N_0 + 3$ configuration going from zero detuning in panel (a), towards stronger detuning in panel (f). The detuning electric field breaks the rotational symmetry and introduces orbital scattering, where the increasing disorder opens, and widens, the window for Zeeman spin splitting along $B_\perp$.

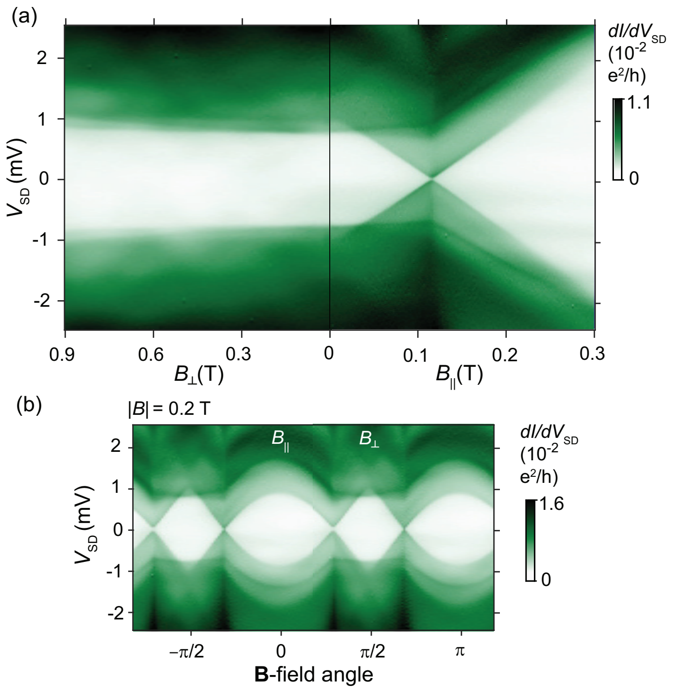

Supplementary FIG 10. Cotunneling spectroscopy in the $N = N_0 + 2$ regime. **(a)** We note that SOI is larger and corresponding exchange energy (split between S± and T*) is smaller compared to the orbital in Fig. 3. **(b)** Rotation of an in-plane $|B| = 0.2$T.

[1] H. Potts, J. Josefi, I.-J. Chen, S. Lehmann, K. A. Dick, M. Leijnse, S. M. Reimann, J. Bengtsson, and C. Thelander, Phys. Rev. B **104**, L081409 (2021).
[2] J. Planelles, F. Rajadell, and J. I. Climente, Nanotechnology **18**, 375402 (2007).
[3] F. E. Meijer, A. F. Morpurgo, and T. M. Klapwijk, Phys. Rev. B **66**, 033107 (2002).
